# Supplementary material for: Diabetes self-care and its associated factors among type 2 diabetes mellitus with chronic kidney disease patients in the East Coast of Peninsular Malaysia
Source: PeerJ. 2024 Oct 17;12:e18303. doi: 10.7717/peerj.18303 (PMC11491061; doi:10.7717/peerj.18303)
Supplement: Supplemental Information 1 [file peerj-12-18303-s001.docx]

## Appendix 4: Case Report Form

**Bahagian A: Maklumat Demografi**

Arahan: Sila tanda (✓) pada kotak yang berkenaan berdasarkan jawapan anda.

1. Umur: ……………..tahun
2. Jantina:

|  | Lelaki |
| --- | --- |
|  | Perempuan |

1. Bangsa:

|  | Melayu |
| --- | --- |
|  | Cina |
|  | India |
|  | Siam |
|  | Lain-lain (sila nyatakan)  …………………………………………. |

1. Status perkahwinan:

|  | Berkahwin |
| --- | --- |
|  | Bujang/Berpisah/Kematian pasangan |

1. Peringkat Pendidikan:

|  | Tidak bersekolah |
| --- | --- |
|  | Sekolah Rendah (UPSR) |
|  | Sekolah Menengah (PMR, SPM, STPM, SMV,STMA) |
|  | Tertiari/Kolej/Universiti (Diploma, Kursus Pengkhususan, Sarjana, Doktor Falsafah, Sub-kepakaran) |

1. Pekerjaan:

|  | Bekerja |
| --- | --- |
|  | Tidak bekerja |

1. Pendapatan isi rumah sebulan: ……………………………….
2. Berapa lama anda menghidapi penyakit kencing manis?

………………. tahun

1. Adakah anda mempunyai sejarah keluarga yang menghidapi kencing manis?

| YA |  | TIDAK |  |
| --- | --- | --- | --- |

1. Adakah anda mempunyai komplikasi penyakit kencing manis lain?

| YA |  | TIDAK |  |
| --- | --- | --- | --- |

1. Adakah anda mempunya penyakit lain selain penyakit kencing manis?

| YA |  | TIDAK |  |
| --- | --- | --- | --- |

1. Apakah cara rawatan kencing manis anda sekarang?

|  | Ubat oral sahaja |
| --- | --- |
|  | Ubat oral dan insulin |
|  | Ubat insulin sahaja |

**Bahagian B: Bahagian ini akan diisi oleh penyelidik berdasarkan rekod perubatan dan pemeriksaan fizikal** **pesakit**

1. Tahap HbA1c:……………(%)
2. Bacaan eGFR: ……………ml/min/1.73m^2^
3. Tinggi:…………m

Berat:………..kg

BMI:………..kg/m^2^

1. Laporan ultrasound buah pinggang:

| Laporan ultrasound | Kiri | Kanan |
| --- | --- | --- |
| Renal Length (mm) |  |  |
| Renal cortical thickness (mm) |  |  |

## Appendix 5: Malay version The diabetes self-care activities (SDSCA) questionnaire

**Bahagian B: Ringkasan Aktiviti Penjagaan Diri Pesakit Kencing Manis (SDSCA)**

Soalan di bawah adalah berkaitan aktiviti anda dalam mengawal penyakit kencing manis sepanjang 7 hari yang lalu. Jika anda sakit sepanjang 7 hari yang lalu, fikirkan 7 hari sebelum anda sakit.

| HARI | 0 | 1 | 2 | 3 | 4 | 5 | 6 | 7 |
| --- | --- | --- | --- | --- | --- | --- | --- | --- |
| **PEMAKANAN:**  Berapa harikah sepanjang **TUJUH HARI** terakhir itu, anda mematuhi pelan pemakanan yang sihat? |  |  |  |  |  |  |  |  |
| Secara purata, sepanjang bulan yang lalu, berapa harikah **DALAM SEMINGGU** anda mematuhi pelan pemakanan sihat anda? |  |  |  |  |  |  |  |  |
| Berapa harikah sepanjang **TUJUH HARI** yang laku anda mengambil hildangan atau lebih yang terdiri daripada buah-buahan dan sayur- sayuran? |  |  |  |  |  |  |  |  |
| Berapa harikah dalam tempoh **TUJUH HARI** yang lalu anda membahagikan pengambilan karbohidrat seperti nasi dan roti secara seimbang dalam satu hari? |  |  |  |  |  |  |  |  |
| HARI | 0 | 1 | 2 | 3 | 4 | 5 | 6 | 7 |
| **SENAMAN:**  Berapa harikah dalam tempoh **TUJUH HARI** yang lalu anda melakukan aktiviti fizikal sekurang-kurangnya 30 minit? (Jumlah masa keseluruhan bagi aktiviti yang dijalankan secara berterusan termasuk berjalan kaki) |  |  |  |  |  |  |  |  |
| Berapa harikah dalam tempoh **TUJUH HARI** yang lalu anda melakukan aktiviti senaman yang khusus (seperti berenang, berjalan, berbasikal) selain daripada aktiviti harian di rumah atau di tempat kerja? |  |  |  |  |  |  |  |  |
| **UJIAN GULA DALAM DARAH:**  Berapa harikah dalam tempoh TUJUH HARI yang lalu anda memeriksa paras gula dalam darah? |  |  |  |  |  |  |  |  |
| Berapa harikah dalam tempoh **TUJUH HARI** yang lalu anda memeriksa paras gula dalam darah mengikut bilangan yang disyorkan oleh doktor/pendidik diabetes anda? |  |  |  |  |  |  |  |  |
| HARI | 0 | 1 | 2 | 3 | 4 | 5 | 6 | 7 |
| Berapa harikah dalam tempoh **TUJUH HARI** yang lalu anda memeriksa bahagian dalam kasut anda? |  |  |  |  |  |  |  |  |
| **MEROKOK:**  Adakah anda pernah menghisap rokok walaupun hanya satu sedutan, sepanjang tempoh **TUJUH HARI** yang lalu? | YA. Jika YA, sila nyatakan berapa purata batang rokok sehari anda hisap | | | | TIDAK | | | |
|  |  | | | |  | | | |

## Appendix 6: Malay version diabetes distress score

**Bahagian C: Versi 17-Perkara Bagi Skala Penderitaan Diabetes (DDS)**

***ARAHAN :*** Hidup berpenyakit kencing manis kadangkala menyusahkan. Terdapat pelbagai masalah dan gangguan berkaitan penyakit kencing manis yang mungkin timbul. Tahap masalahnya mungkin sangat berbeza, iaitu daripada gangguan kecil hingga membawa kesengsaraan teruk. Di bawah ini, tersenarai 17 perkara yang mungkin dialami oleh pensakit kencing manis. Pertimbangkan tahap gangguan bagi setiap 17 perkara tersebut sepanjang **BULAN LEPAS** dan bulatkan nombor yang sesuai.

Sila ambil perhatian bahawa kami memerlukan anda untuk memberitahu tahap gangguan setiap perkara yang berlaku dalam kehidupan anda, **BUKAN** sahaja perkara itu benar-benar berlaku pada diri anda. Jika anda berasa bahawa perkara tersebut bukan gangguan atau masalah untuk anda, bulatkan “**1**”. Jika perkara itu sangat mengganggu hidup anda, bulatkan "**6**".

| **No.** | ***Masalah-masalah*** | Bukan Masalah | Masalah Kecil | Masalah Sederhana | Masalah Agak Serius | Masalah Serius | Masalah Sangat Serius |
| --- | --- | --- | --- | --- | --- | --- | --- |
|  | Berasa bahawa doktor saya tidak cukup mengetahui penyakit kencing manis dan cara menanganinya. | 1 | 2 | 3 | 4 | 5 | 6 |
|  | Berasa bahawa kerana penyakit kencing manis, saya kehilangan terlalu banyak tenaga mental dan fizikal setiap hari. | 1 | 2 | 3 | 4 | 5 | 6 |
|  | Tidak berasa yakin dengan keupayaan saya untuk mengendalikan penyakit kencing manis setiap hari. | 1 | 2 | 3 | 4 | 5 | 6 |
| **No.** | ***Masalah-masalah*** | Bukan Masalah | Masalah Kecil | Masalah Sederhana | Masalah Agak Serius | Masalah Serius | Masalah Sangat Serius |
|  | Berasa marah, takut dan/atau tertekan apabila saya memikirkan hidup dengan penyakit kencing manis. | 1 | 2 | 3 | 4 | 5 | 6 |
|  | Berasa bahawa doktor saya tidak memberikan arahan yang cukup jelas tentang cara mengendalikan penyakit kencing manis saya. | 1 | 2 | 3 | 4 | 5 | 6 |
|  | Berasa bahawa saya tidak menguji kandungan gula dalam darah dengan cukup kerap. | 1 | 2 | 3 | 4 | 5 | 6 |
|  | Berasa bahawa saya akan mengalami komplikasi jangka panjang yang serius, tidak kira apa-apa pun yang saya lakukan. | 1 | 2 | 3 | 4 | 5 | 6 |
|  | Berasa bahawa saya sering gagal dengan rutin pengawalan penyakit kencing manis saya. | 1 | 2 | 3 | 4 | 5 | 6 |
| **No.** | ***Masalah-masalah*** | Bukan Masalah | Masalah Kecil | Masalah Sederhana | Masalah Agak Serius | Masalah Serius | Masalah Sangat Serius |
|  | Berasa bahawa rakan atau keluarga tidak memberikan sokongan secukupnya dalam usaha penjagaan diri (misalnya merancang aktiviti yang bercanggah dengan jadual saya, menggalakkan saya untuk makan makanan yang "salah"). | 1 | 2 | 3 | 4 | 5 | 6 |
|  | Berasa bahawa penyakit kencing manis mengawal hidup saya. | 1 | 2 | 3 | 4 | 5 | 6 |
|  | Berasa bahawa doktor saya tidak memberikan perhatian yang cukup serius terhadap kebimbangan saya. | 1 | 2 | 3 | 4 | 5 | 6 |
| **12.** | Berasa bahawa saya tidak mematuhi pelan pemakanan yang baik sepenuhnya. | 1 | 2 | 3 | 4 | 5 | 6 |
| **No.** | ***Masalah-masalah*** | Bukan Masalah | Masalah Kecil | Masalah Sederhana | Masalah Agak Serius | Masalah Serius | Masalah Sangat Serius |
| **13.** | Berasa bahawa rakan atau keluarga tidak dapat memahami kesengsaraan hidup yang mungkin dialami oleh penghidap kencing manis. | 1 | 2 | 3 | 4 | 5 | 6 |
| **14.** | Berasa terbeban untuk menjalani hidup dengan berpenyakit kencing manis. | 1 | 2 | 3 | 4 | 5 | 6 |
| **15.** | Berasa bahawa saya tidak mempunyai doktor yang dapat ditemui dengan kerap untuk berbincang tentang penyakit kencing manis saya. | 1 | 2 | 3 | 4 | 5 | 6 |
| **16.** | Berasa tidak bermotivasi untuk terus mengurus penyakit kencing manis dengan sendiri. | 1 | 2 | 3 | 4 | 5 | 6 |
| **17.** | Berasa bahawa rakan atau keluarga tidak memberi saya sokongan emosi yang diingini. | 1 | 2 | 3 | 4 | 5 | 6 |

## Appendix 7: Patient Health Questionnaire

**Bahagian D: Soal Selidik Kesihatan Pesakit (PHQ-9)**

Soal-selidik ini amat penting untuk menilai tahap kesihatan anda. Jawapan anda akanmembantu untuk memahami masalah yang mungkin anda hadapi:

Dalam 2 minggu lepas, berapa banyak kalikah, anda berasa terganggu, oleh mana-mana sebab dibawah? Tandakan (X) pada yang berkenaan

|  | **Tidak langsung**  **0** | **Beberapa Hari**  **1** | **Lebih daripada seminggu**  **2** | **Hampir setiap hari**  **3** |
| --- | --- | --- | --- | --- |
| 1. Kurang berminat atau kenikmatan dalam membuat sesuatu perkara |  |  |  |  |
| - 1. Muram, tertekan atau putus harapan |  |  |  |  |
| - 1. Sukar untuk tidur atau tidur berlebihan |  |  |  |  |
| - 1. Kepenatan atau kurang tenaga |  |  |  |  |
| - 1. Hilang selera atau makan berlebihan |  |  |  |  |
| - 1. Berasa diri sendiri amat teruk - atau berasa anda orang yang gagal atau telah mengecewakan diri sendiri atau keluarga |  |  |  |  |
| - 1. Sukar untuk beri tumpuan kepada sesuatu seperti membaca suratkhabar atau menonton televisyen. |  |  |  |  |
| - 1. Sama ada bergerak atau bercakap dengan perlahan sehingga orang lain pun dapat menyedarinya - atau sebaliknya - menjadi gugup atau gelisah atau membuat pergerakan yang lebih dari biasa. |  |  |  |  |
| - 1. Berfikiran bahawa mati adalah lebih baik daripada terus hidup atau ingin mencederakan diri dengan cara apa sekalipun. |  |  |  |  |
