# Supplementary material for: Diabetes self-care and its associated factors among type 2 diabetes mellitus with chronic kidney disease patients in the East Coast of Peninsular Malaysia
Source: PeerJ. 2024 Oct 17;12:e18303. doi: 10.7717/peerj.18303 (PMC11491061; doi:10.7717/peerj.18303)
Supplement: Supplemental Information 2 [file peerj-12-18303-s002.docx]

**Section A: Demographic**

Instructions: Please tick (✓) the appropriate box based on your answer..

1. Age: ……………..years old
2. Gender:

|  | Male |
| --- | --- |
|  | Female |

1. Race:

|  | Malay |
| --- | --- |
|  | Chinese |
|  | Indian |
|  | Siamese |
|  | Others: please state  …………………………………………. |

1. Marital status:

|  | Married |
| --- | --- |
|  | Single/Separation/Death of spouse |

1. Level of Education:

|  | None |
| --- | --- |
|  | Primnary school (UPSR) |
|  | Secondary school (PMR, SPM, STPM, SMV,STMA) |
|  | Tertiary/College/University (Diploma, Specialization Course, Masters, Doctor of Philosophy, Sub-specialization) |

1. Employment:

|  | Employed |
| --- | --- |
|  | Unemployed |

1. Monthly household income: ……………………………….
2. How long have you had diabetes?

………………. years

1. Do you have a family history of diabetes?

| YES |  | NO |  |
| --- | --- | --- | --- |

1. Do you have other complications of diabetes?

| YES |  | NO |  |
| --- | --- | --- | --- |

1. Do you have other diseases besides diabetes?

| YES |  | NO |  |
| --- | --- | --- | --- |

1. What is your diabetes treatment now??

|  | Oral only |
| --- | --- |
|  | Oral dan insulin |
|  | Insulin only |

**Section B: This part will be filled out by the researcher based on the patient's medical records and physical examination**

1. HbA1c:……………(%)
2. eGFR: ……………ml/min/1.73m^2^
3. Heighti:…………m

Wight:………..kg

BMI:………..kg/m^2^

1. Kidney ultrasound report:

| Ultrasound report | Left | Right |
| --- | --- | --- |
| Renal Length (mm) |  |  |
| Renal cortical thickness (mm) |  |  |

**Section C: The diabetes self-care activities (SDSCA) questionnaire**

The questions below are related to your activities in controlling diabetes over the past 7 days. If you have been sick for the past 7 days, think back to the 7 days before you were sick.

| DAYS | 0 | 1 | 2 | 3 | 4 | 5 | 6 | 7 |
| --- | --- | --- | --- | --- | --- | --- | --- | --- |
| **FOOD:**  How much of the last SEVEN DAYS did you stick to a healthy eating plan? |  |  |  |  |  |  |  |  |
| On average, over the past month, how many days PER WEEK did you stick to your healthy eating plan? |  |  |  |  |  |  |  |  |
| How often during SEVEN DAYS do you eat a meal or more consisting of fruits and vegetables? |  |  |  |  |  |  |  |  |
| How often in the past SEVEN DAYS did you divide your intake of carbohydrates such as rice and bread evenly in one day? |  |  |  |  |  |  |  |  |
| **EXERCISE:**  How many days in the past SEVEN DAYS did you do physical activity for at least 30 minutes? (Total amount of time for activities carried out continuously including walking) |  |  |  |  |  |  |  |  |
| How many times in the past SEVEN DAYS did you do specific exercise activities (such as swimming, walking, cycling) other than your daily activities at home or at work? |  |  |  |  |  |  |  |  |
| **BLOOD SUGAR TEST:**  How often in the past SEVEN DAYS did you check your blood sugar level? |  |  |  |  |  |  |  |  |
| How many times in the last SEVEN DAYS did you check your blood sugar level according to the number recommended by your doctor/diabetes educator? |  |  |  |  |  |  |  |  |
| How often in the last SEVEN DAYS did you check the inside of your shoes? |  |  |  |  |  |  |  |  |
| **SMOKING:**  Have you ever smoked a cigarette, even just one puff, during the past SEVEN DAYS? | YES. If YES, please indicate the average number of cigarettes you smoke per day | | | | NO | | | |
|  |  | | | |  | | | |

**Section D: THE DIABETES DISTRESS SCREENING SCALE**

***Directions :*** Living with diabetes can sometimes be touch. There may be many problems and hassles concerning diabetes and they can vary greatly in severity. Problems may range from minor hassles to major life difficulties. Listed below are 17 potential problem areas that people with diabetes experience. Consider the degree to which each of the 17 items may have distressed or bothered you DURING THE PAST MONTH and circle the appropriate number.

Please note that we are asking you to indicate the degree to which each item may be bothering you in your life, NOT whether the item is merely true for you. If you feel that a particular item is not a bother or a problem for you, you would circle “1. If it is bohersome to you, you might circle “6”.

| **No.** | ***Problems*** | Not a problem | A slight problem | A moderate problem | Somewhat serious problem | A serious problem | A very serious problem |
| --- | --- | --- | --- | --- | --- | --- | --- |
|  | Feeling that my doctor doesn't  know enough about diabetes and diabetes care. | 1 | 2 | 3 | 4 | 5 | 6 |
|  | Feeling that diabetes is taking up too much of my mental and physical energy every day. | 1 | 2 | 3 | 4 | 5 | 6 |
|  | Feeling that I am often failing with my diabetes routine. | 1 | 2 | 3 | 4 | 5 | 6 |
|  | Feeling angry, scared, and/or depressed when I think about living with diabetes. | 1 | 2 | 3 | 4 | 5 | 6 |
|  | Feeling that my doctor doesn't  give me clear enough directions on how to manage my diabetes. | 1 | 2 | 3 | 4 | 5 | 6 |
|  | Feeling that I am not testing my blood sugars frequently enough. | 1 | 2 | 3 | 4 | 5 | 6 |
|  | Feeling that friends or family are not supportive enough of self-care efforts( eg planning activities that conflict with my schedule, encouraging me to eat the “wrong” foods) | 1 | 2 | 3 | 4 | 5 | 6 |
|  | Feeling that diabetes controls my life | 1 | 2 | 3 | 4 | 5 | 6 |
|  | Feeling that my doctor doesn’t take my concerns seriously enough | 1 | 2 | 3 | 4 | 5 | 6 |
|  | Not feeling confident in my day-to-day ability to manage diabetes | 1 | 2 | 3 | 4 | 5 | 6 |
|  | Feeling that I will end up with serious long-term complication no matter what I do. | 1 | 2 | 3 | 4 | 5 | 6 |
| **12.** | Feeling that I am not sticking closely enough to a good meal plan | 1 | 2 | 3 | 4 | 5 | 6 |
| **13.** | Feeling that friends and family don’t appreciated how difficult living with diabetes can be | 1 | 2 | 3 | 4 | 5 | 6 |
| **14.** | Feeling overwhelmed by the demands of living with diabetes. | 1 | 2 | 3 | 4 | 5 | 6 |
| **15.** | Feeling that I don’t have a doctor who can see regularly enough about my diabetes | 1 | 2 | 3 | 4 | 5 | 6 |
| **16.** | Not feeling motivated to keep up my diabetes self management | 1 | 2 | 3 | 4 | 5 | 6 |
| **17.** | Feeling that friends or family don’t give me the emotional support that I would like | 1 | 2 | 3 | 4 | 5 | 6 |

**Section E: Patient Health Questionnaire (PHQ-9)**

Over the last 2 weeks, how often have you been bothered by any of the following problems?

(use X to indicate your answer)

|  | **Not at all**  **0** | **Several days**  **1** | **More than half the days**  **2** | **Nearly every day**  **3** |
| --- | --- | --- | --- | --- |
| 1. Little interest or pleasure in doing things |  |  |  |  |
| - 1. Feeling down, depressed or hopeless |  |  |  |  |
| - 1. Trouble falling or staying asleep or sleeping too much |  |  |  |  |
| - 1. Feeling tired or having little energy |  |  |  |  |
| - 1. Poor appetite or overeating |  |  |  |  |
| - 1. Feeling bad about yourself or that you are a failure or have let yourself or your family down |  |  |  |  |
| - 1. Trouble concentrating on things, such as reading the newspaper or watching television |  |  |  |  |
| - 1. Moving or speaking so slowly that other people could have noticed. Or the opposite being so figety or restless that you have moving around a lot more than usual |  |  |  |  |
| - 1. Thoughts that you would be better off dead, or of hurting yourself |  |  |  |  |
